# Honoring Long-Lived Cultural Beliefs for End-of-Life Care: Are We Prepared in the Modern Western Society?

**DOI:** 10.7759/cureus.30313

**Published:** 2022-10-14

**Authors:** Kevin Wong, James H. S. Camacho, Saakshi Dulani, Kovid Trivedi

**Affiliations:** 1 Internal Medicine, Western University of Health Sciences, Lebanon, USA; 2 Pulmonary/Critical Care Medicine, Salem Pulmonary Associates/Salem Health, Salem, USA

**Keywords:** attitude to death, death and dying, cultural sensitivity, religious perspective, helipad, hospice and palliative care, terminal extubation, team based end of life care

## Abstract

The Hippocratic Oath establishes the principle of *primum non nocere* or "first do no harm" in Western medicine. This not only includes physical health but also encompasses emotional and spiritual health. Various end-of-life care (EOLC) practices exist in different societies, and it is hard but vital for the healthcare community to be aware of these practices to allow wholesome care for their patients, which is emotionally and spiritually fulfilling.

A 57-year-old male with a history of metastatic squamous cell carcinoma of the head and neck region presented to the emergency department after an out-of-hospital cardiac arrest. After appropriate post-cardiac arrest care, the patient remained unresponsive, and the family decided to transition to comfort-focused care. Based on their religious and cultural preferences, they preferred palliative extubation at a place where the patient would not have a roof over his head at the time of death, as well as sought help to facilitate a same-day funeral. After coordinating with various departments in the hospital, the patient was taken to the hospital helipad and extubated there in the family's presence. The patient's remains were released to the family within an hour of death for a timely funeral.

This case is an example of cultural and religious diversity that exists within our community. Healthcare is a complex field and EOLC is a crucial part of patient care. With a multi-disciplinary approach towards EOLC, the distress related to death can be reduced among families as well as healthcare teams.

## Introduction

*Primum non nocere* is the Latin of “first do no harm” in the original Hippocratic Oath. This oath is one of the earliest expressions of bioethics in Western medical literature and continues to be one of the integral tenets of modern medicine. Not only does this principle pertain to the physical wellbeing of patients but also to their spiritual and emotional wellbeing. In medicine, all these aspects of patient care are touched upon at the end of a patient's life. End-of-life care (EOLC) is a shift from prolongation of life to symptom control, comfort, dignity, quality of death, and after-death care. Patients, nearing the end of their life, typically receive care in four main areas: mental and emotional care, physical care, spiritual care, and practical tasks. Depending on the cultural and religious practices of the patient, EOLC is different when honoring the needs of the dying [1]. In addition, families and communities of the dead have different traditions and cultures affecting EOLC.

In an ever-growing multicultural society, cultural competency and sensitivity are necessities for EOLC but can be easily compromised by the care teams’ lack of knowledge or hospital system-related barriers, the latter referring to facilities lacking amenities for cultural diversity and favoring a universal approach to EOLC. Barriers within the hospital system place a limit on materials and personnel, preventing the facility's ability to adjust patient care in their approach to death and dying [2]. For places such as the intensive care unit (ICU), many accommodations cannot be met due to logistics and acuity of patient care, and often lead to undesired outcomes and interactions. Arguably, limitations on EOLC violate the notion of non-maleficence and beneficence due to the inability of healthcare providers to care for the emotional and spiritual wellbeing of their patients. This ethical dissonance calls attention to the difficulty that the healthcare team in the ICU must face in providing holistic and quality care to their patients as well as highlights the need for our healthcare systems to account for cultural, religious, and spiritual diversity. We present a case where the routine functioning of the health system was modified to accommodate the religious and cultural practices of the patient and the family.

## Case presentation

A 57-year-old male was brought to the emergency department (ED) after an out-of-hospital cardiac arrest. The family found him unresponsive on his bed; he was last seen normal about an hour ago. Bystander cardiopulmonary resuscitation (CPR) was done by the family for about 10 minutes before the paramedics arrived. The patient had a return of spontaneous circulation at the time of the paramedics’ arrival. En route to the hospital, the patient had repeated episodes of cardiac arrest, with a cumulative estimated arrest time of 25 minutes. The patient had a history of primary head and neck cancer (status post partial glossectomy and lymph node dissection, followed by chemoradiation), followed by metastatic recurrence, for which he was on immunotherapy. Past medical history was also significant for type II diabetes mellitus, hypertension, hepatitis C, coronary artery disease, unilateral below-knee amputation, renal cell carcinoma (status post partial nephrectomy), and dysphagia. The patient was admitted to the ICU and treated with targeted temperature management (TTM), broad-spectrum antibiotics, and mechanical ventilatory support. Over the hospital course, the patient had poor neurological recovery and went into status epilepticus after rewarming and cessation of sedation. Aggressive management of seizures was done. Family meetings were held for goals of care discussion based on poor neurological prognosis in a patient with multiple medical problems. The family decided to proceed with transition to comfort-focused care.

Based on their cultural and religious practices, the family preferred at-home extubation with a request for the absence of a roof over the patient’s head at the time of demise. Cultural preference also demanded burial before sun-down the same day. Multiple hospice agencies were contacted, and they expressed a lack of support to facilitate at-home extubation. After further engagement, the family agreed to palliative extubation in the hospital. To honor the patient’s and family’s religious/cultural beliefs, the patient was taken to the helipad of the hospital (after clearance from ED, security, and emergency flight crew) and the palliative extubation was performed with no roof over his head. In the presence of the family at the bedside during that time, the patient had a peaceful demise. In coordination with the hospital personnel, the remains of the patient were released to the family within the next hour to facilitate a same-day funeral. The religious and cultural practices were not verified due to the sensitive nature of the topic and to support the spiritual well-being of the family.

## Discussion

Various EOLC practices exist among different cultures and religions, which have also changed based on present world scenarios. Difficulties arise in hospitals when the staff is not familiar with these practices among minority populations.

People from all over the world observe funeral traditions and burial rites in ways that are unique to their cultures. Despite the various ways to honor a peaceful death, core fundamental values in the human society like community, collaboration, and respect for the dead remain evident across cultures. In Haiti, both Catholic and West-African customs are followed simultaneously. Death is viewed as a social affair to celebrate the life of the deceased. Over the course of nine days, family, friends, and neighbors sing songs, play music, share food, and pray, before the body is finally laid to rest [3]. The nine-day celebration of "waking the dead" is also followed in the Philippines, and this funeral tradition highlights the native people’s belief in the continuum of life after death and that with community, even the toughest hardships become bearable [4]. In Samoa, people celebrate death through economic and communal reciprocity where family members are contacted from overseas for both material and monetary donations as part of the funeral arrangement process [5]. In some Buddhist cultures, the dying repeat the names of Buddha or have someone whisper Buddhist scriptures or the names of Buddha in their ear if they are unable to speak [6]. In Hinduism, signs of impending death begin with the chanting of holy scriptures and putting holy water from the Ganges River and *Tulsi* leaves (Indian basil) in the mouth of the dying. The body is then prepared for same-day or next-day cremation while family members initiate the cremation fire and prefer to observe the cremation [7]. In the Muslim faith, the face of the deceased is turned toward Mecca (Ka'bah), the arms and legs are straightened, the eyes and jaw closed, all clothing is removed by a Muslim person of the same sex, and the deceased is then covered with a sheet. The body is then bathed by respected elders of the Muslim faith, preferably the same sex as the deceased, and clothed in white. Family members of the deceased are responsible for all transportation and immediate burial following death [8]. 

## Conclusions

Honoring death in accordance with *primum non nocere* begins with the family and asking questions about cultural beliefs and practices unique to death and funeral rites. With the collaboration between the family of the deceased and the hospital staff, certain provisions can be made to allow the family to spend more time with the deceased and perform last rites necessary to the family’s culture, contact other family members to be involved in the funeral arrangement process, and educate hospital staff about diversity, equity, and inclusion from a palliative care standpoint. Just like how providing a roofless environment for our patient in this case meant conducting terminal extubation at the helipad of the hospital, this type of work involves the entire hospital brainstorming different ways that our healthcare community can help accommodate these unique approaches to EOLC. This improves the overall patient and family experience from an emotional, mental, religious, and spiritual perspective. By creating a system where we can account for diversity in EOLC, we can better provide holistic, quality, and comfortable care to patients who are nearing the end of their life.
